# Significance of Cellular Lipid Metabolism for the Replication of Rotaviruses and Other RNA Viruses

**DOI:** 10.3390/v16060908

**Published:** 2024-06-04

**Authors:** Ulrich Desselberger

**Affiliations:** Department of Medicine, University of Cambridge, Addenbrooke’s Hospital, Cambridge CB2 0QQ, UK; ud207@medschl.cam.ac.uk; Tel.: +44-1223-763403; Fax: +44-1223-336846

**Keywords:** lipid metabolism, RNA virus replication, rotavirus, hepatitis C virus, enteroviruses, SARS-CoV-2, HIV-1

## Abstract

The replication of species A rotaviruses (RVAs) involves the recruitment of and interaction with cellular organelles’ lipid droplets (LDs), both physically and functionally. The inhibition of enzymes involved in the cellular fatty acid biosynthesis pathway or the inhibition of cellular lipases that degrade LDs was found to reduce the functions of ‘viral factories’ (viroplasms for rotaviruses or replication compartments of other RNA viruses) and decrease the production of infectious progeny viruses. While many other RNA viruses utilize cellular lipids for their replication, their detailed analysis is far beyond this review; only a few annotations are made relating to hepatitis C virus (HCV), enteroviruses, SARS-CoV-2, and HIV-1.

## 1. Introduction

While the replication cycles of many RNA and DNA viruses have been well researched, it has only relatively recently been recognized that various cellular lipids are involved in many of the viral replication steps [1,2,3,4,5,6,7].

Cellular lipids, collectively termed the ‘liposome’, are classified as membrane lipids (phospholipids, sphingolipids, glycolipids), cholesterol, steroids, triacylglycerol (TAG), fatty acids, and eicosanoids [8]. Viruses interact with host cell lipids in various ways, affecting their biosynthesis and metabolism and gaining energy for their own replication [3]. In this study, the significance of cellular lipids for the replication of rotaviruses will be reviewed in some detail. Analogous data have been acquired for many other RNA viruses. The fact that the disturbance of cellular lipid metabolism decreases the yield of infectious viral progeny for many viruses has made the search for lipid-disturbing compounds an attractive aim for the development of broadly active candidate antivirals [1,5,6,7,9].

## 2. Rotaviruses

**Figure 1:**
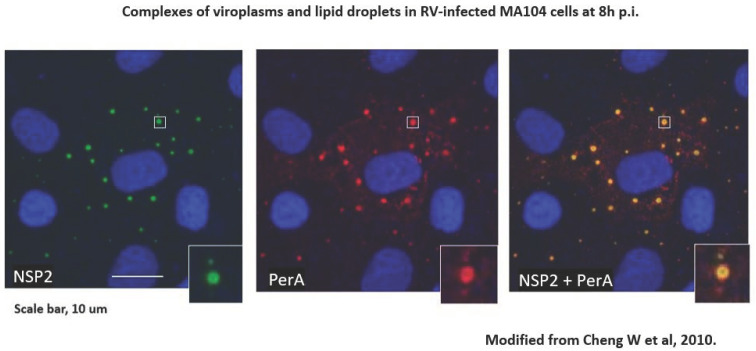
RV-infected cells grown on coverslips were processed for confocal microscopy (CM) as described [20]. Cells were blocked with 1% BSA–0.1% triton X-100. Primary antibodies against perilipin A (rabbit polyclonal) were from Abcam, and those against NSP2 (mouse monoclonal) were a kind gift of Dr Oscar Burrone, ICGEB, Trieste IT. Secondary antibodies were goat anti-rabbit IgG conjugated with Alexafluor 633 and goat anti-mouse IgG conjugated with Alexafluor 488, both from Invitrogen. Staining with secondary antibodies was carried out in the presence of 2 µg/mL of Hoechst 33,342 (from Sigma). Coverslips were mounted on glass slides with Prolong bold Antifade mounting medium (from Molecular Probes) and observed by CM using a Leica DM Libre TCS SP instrument. An individual viroplasm–LD complex is magnified in the inserts. From [17].

Species A rotaviruses (RVAs) are a major cause of severe gastroenteritis in infants and young children worldwide and in the young of many mammalian and avian host species [10]. RVA-associated disease leads to the death of approximately 130,000 children under the age of 5 y annually worldwide, mainly in low-income countries [11]. Two RVA vaccines have been licensed since 2006 and are now used in expanded programs of immunization (EPIs) in over 100 countries [12]. In addition, alternative RV vaccines were licensed in India, Vietnam, and China [12]. RVA EPIs have significantly decreased RVA-associated disease, although at different levels in different parts of the world [13,14]. The molecular biology of RVA replication has been well studied [10]. During replication, ‘viroplasms’ form, which are intracytoplasmic inclusion bodies utilized for early RVA morphogenesis and viral RNA replications to take place; they have been termed ‘viral factories’ and have been recognized as essential [10]. The rotavirus-encoded components of viroplasms, NSP2 and NSP5, are subject to liquid–liquid phase separation [15,16]. In 2010, rotavirus viroplasms (containing VP1, VP2, VP3, VP6 and viral RNA segments) were discovered to be associated with components of cellular organelles’ lipid droplets (LDs) [17] [Figure 1]. LDs of various sizes (0.05–200 µm in diameter) are storage sites of sterol esters and triglycerides, which serve as major cellular energy sources and are in close contact with the mitochondria and the 65 endoplasmic reticulum (ER). The phospholipid monolayer of LDs contains various proteins such as perilipins [PLINs], diacylglycerol acyl transferase 2 [DGAT2], Rab18 [a transport protein], CTP-phosphocholine cytidylyltransferase [CCT], the rate-limiting enzyme of phosphatidylcholine synthesis [18], and various other proteins such as ADRP adipophilin and tip47, often bound by amphipathic helices [18]. LDs also contain acyl-CoA cholesterol acyltransferases 1 and 2 (ACAT1, ACAT2), which catalyze cholesterol esterification. A genome-wide, siRNA- based screen identified 550/18,000 genes of human macrophages (THP-1) as being involved in modulating lipid storage according to number, size, and cellular localization and functions such as proteasome activity, intracellular transport, transcription activity, E3 ligase activity, and lipid-modifying enzymes [19]. In summary, LDs are central to the regulation of cellular lipid homeostasis [19]. Fluorescence resonance energy transfer (FRET) has been shown to occur between perilipin A and NSP2, proving the close spatial proximity of LDs and viroplasms [17].

The iodixanol gradient velocity ultracentrifugation of RV-infected cell extracts resulted in the co-sedimentation of viral dsRNA, NSP2, and perilipin A in low-density fractions (1.11–1.15 g/mL). In contrast, rotavirus dual-layered particles (DLPs, with a density of 1.38 g/mL) spiked into mock-infected lysates sedimented to the bottom of the gradient [17]. Iodixanol gradient fractions of low density, containing peaks of the RV dsRNA genome and LD- and viroplasm-associated proteins, were analyzed for 14 different classes of lipids by mass spectrometry and found to contain maximum amounts of lipids as typical components of LDs, confirming the close interaction of LDs with viroplasms [21]. The molecular mechanisms underlying the complex formation of LDs and viroplasms are underexplored [16]. The viral NSP5 protein, an essential component of viroplasms, contains an amphipathic helix [22], which possibly mediates the interaction with LDs, as do other cellular proteins containing amphipathic helices [23].

The complex formation of viroplasms and LDs has functional consequences. LD formation depends critically on the presence of long-chain fatty acids. The following enzymes contribute to their biosynthesis: acetyl-CoA cocarboxylase (ACC-1), fatty acid synthase (FASN), long-chain acyl-CoA synthetase (HCSL), and various glycerol-acyl transferases (GATs), the latter converting long-chain fatty acids into triacyl esters (neutral fats), which are incorporated into the precursors of LDs in the endoplasmic reticulum (ER). Lipid droplets bud from the lipid bilayer of the ER into the cytoplasm and acquire various lipid droplet-associated proteins (PLINs 1–5 and many others) [2].

The key inhibitors of these pathways are TOFA (inhibiting ACC-1), C75 (anti-FASN), triacsin C (anti-ACSL), and A922500 (anti-DGAT-1) [2,24]. The application of these inhibitors to RV-infected cells significantly reduces the size and number of viroplasms formed, the production of viral dsRNA, and the titers of infectious viral progeny. Similarly, the treatment of cells with a combination of [isoproterenol + IBMX], which fragments LDs into smaller microdroplets, also reduces RV progeny production. Representative data for inhibition with TOFA, triacsin C, and [isoproterenol + IBMX] are presented in Table 1 [17,25]. The inhibitory effects of those compounds were reproducibly recorded at non-cytotoxic concentrations; the off-target effects of the compounds cannot be fully excluded at this stage. 

Furthermore, TOFA-treated and RV-infected cells were analyzed for the production of DLPs and TLPs (purified by CsCl gradient equilibrium ultracentrifugation). In the presence of TOFA, the production of DLPs decreased by a factor of two, but that of TLPs decreased by a factor of 20 compared to the DLP and TLP production in the untreated cells. This suggests that the inhibition of fatty acid biosynthesis affects not only the recruitment of LDs by viroplasms but is also involved in interfering with the later stages of RV maturation [26]. The molecular mechanism is not clear but possibly involves alterations of intracellular membranes, which provide transient envelopes during particle maturation in the cytoplasm [27]. The mechanism of the interaction of viroplasms with LDs was explored further. Following cellular infection with an RVA mutant with delayed viroplasm formation (rRV NSP2 S313D, engineered by a RVA-specific, plasmid only-based reverse genetics system; according to [28]), an early interaction of viroplasm-bound NSP2 with phospho-perilipin 1 (leading to lipolysis [29]) was observed [30], thus exploiting the lipid metabolism.

Recently, it was found that DGAT1, required for triacylglycerol and LD biosynthesis, is degraded in RV-infected MA104 cells and in human intestinal enteroids (HIEs) [31]. In an uninfected cell, DGAT1 synthesizes TAGs from ER-resident DAGs and cytoplasmic acyl-CoAs, and TAGs are deposited in the ER lipid bilayer. Upon RV infection, NSP2 is expressed, which interacts with DGAT1. The viroplasm–DGAT1 complexes are then tagged with ubiquitin and degraded in proteasomes. The loss in DGAT1 induces the budding of TAGs and LD formation through a mechanism that is still not fully understood [31]. The suppression of DGAT1 by rotavirus infection leads to an increase in the number of viroplasms and in the infectivity titer of viral progeny in MA104 cells [31].

## 3. Other RNA Viruses

Many RNA viruses other than rotaviruses utilize components of the cellular lipidome for their replication. A detailed analysis of the molecular mechanisms involved is beyond the capacity of this review. In the following, a few observations are noted (Table 2) aimed at emphasizing the great significance of this research.

### 3.1. Hepatitis C Virus

The infection of liver cells with the hepatitis C virus (HCV) was shown to induce cytoplasmic double-membrane vesicle (DMV) compartments, also termed vesicle packets, where major steps of viral replication occur [32]. The viral non-structural protein NS5B interacts with the surface of LDs and, together with oter viral core proteins, is involved in viral RNA replication [32,33]. The protein NS5A binds to LDs via an amphipathic helix (similar to viperin), and LD lipolysis is associated with HCV replication, although the molecular mechanism is not clear yet [34]. While the temporal dynamics of cellular lipid species have been explored by mass spectrometry at early and late time points of HCV infection, the mechanisms correlating HCV replication with lipid dynamics remain to be explored [35].

### 3.2. Enteroviruses and Other Picornaviruses

Phosphatidyl inositole (PI) and its mono- and diphosphate derivatives (PI4P and PI(4.5)P2, respectively) play key roles in the establishment of replication complexes of enteroviruses and other (+)ssRNA viruses [37]. Lipolysis is a major component of enterovirus RNA replication [36,40,41].

### 3.3. SARS-CoV-2

For coronaviruses, mainly SARS-CoV-2, the causative agent of COVID-19, ceramide plays a central role during viral cell entry. Inhibitors of acid sphingomyelinase, the rate-limiting enzyme of ceramide biosynthesis, suppress viral replication [9].

### 3.4. HIV-1

Based on previous findings that ceramide is required for capsid maturation in HIV-1, it was recently observed that the inhibition of neutral sphingomyelinase 2, the rate-limiting enzyme of ceramide biosynthesis, prevented the full maturation of HIV-1 capsids and full infectivity in vitro [38] and in vivo [39].

## 4. Compounds Interfering with Lipidome Homeostasis

The cellular lipidome is beginning to be considered a ‘therapeutic target’ [9] against RNA virus replication. Thus, some compounds interfering with cellular lipidome metabolism are being explored with the aim of reducing viral replication (Table 3).

The HCV NS5A protein, bound to the surface of LDs, interacts with cellular cyclophilin A (CypA), and CypA inhibitors were discovered to block viral RNA replication in HCV-infected cells [42].

Enterovirus replication complexes depend on the lipolysis of triglycerides by hormone-dependent lipase (HDL) or adipose triglyceride lipase (ATGL). In the presence of CAY10499, an HDL inhibitor, or atglistatin, an ATGL inhibitor, enterovirus replication decreased [36].

Against SARS-CoV-2 replication, various drugs reducing the cholesterol synthesis and esterification, LD formation, and ceramide biosynthesis of infected cells are under investigation as potential antivirals [7,9,43].

PDDC, an inhibitor of neutral sphingomyelinase 2 that prevents ceramide biosynthesis, was shown to block the maturation and full infectivity of HIV-1 particles [38,39].

## Figures and Tables

**Table 1:** Comparison of inhibitory effects of different compounds affecting lipid droplet homeostasis on rotavirus replication.

Treatment of Cells		Viral dsRNA		Infectivity of Progeny	
		Relative Value ^a^	Difference ^b^	log TCID_50_/mL ±S.E. (n)	Difference ^b^
Isoproterenol + IBMX ^c^	−	1.00		8.2 ± 0.3 (3)	
	+	0.25	4.0-fold	6.5 ± 0.1 (3)	50-fold
Triacsin C ^c^	−	1.00		7.5 ± 0.1 (3)	
	+	0.26	3.8-fold	6.2 ± 0.2 (3)	20-fold
TOFA ^d^	−	1.00		8.4 ± 0.5 (6)	
	+	0.17	5.9-fold	6.7 ± 0.5 (6)	50-fold

^a^ Calculated from densitometric values of RNA gels [17]. ^b^ All differences shown are statistically significant (*p* < 0.05). ^c^ From [17]. ^d^ From [25].

**Table 2:** Lipid metabolism involved in the replication of some RNA viruses other than rotaviruses.

Virus	Mechanism	References
Hepatitis C virus	Viral replication in double-membrane vesicles: NS5A protein binding to LD surface and interacting with cyclophilin A	[32,33,34]
	Correlation of temporal dynamics of lipids with HCV replication steps is underexplored	[35]
Enteroviruses	Phosphatidyl inositole phosphates (PI4P, PI(4,5)P2) are involved in the establishment of viral replication complexes. Correlation of lipolysis with viral RNA replication	[36,37]
SARS-CoV-2	Central role of ceramide in viral cell entry	[9]
HIV-1	Central role of ceramide for viral capsid maturation in vitro and in vivo	[38,39]

**Table 3:** Compounds disturbing cellular lipidome homeostasis as potential candidate antivirals.

Virus	Compounds	References
Rotavirus	TOFA; [isoproterenol + IBMX]	[2,17]
Hepatitis C virus	Cyclophilin inhibitors blocking viral RNA replication	[42]
Enterovirus	CAY10499 (hormone-dependent lipase inhibitor) and atglistatin (adipose triglyceride lipase inhibitor), both reducing viral replication	[36]
SARS-CoV-2	drugs reducing cholesterol synthesis and esterification, LD formation, and ceramide biosynthesis	[7,9,43]
HIV-1	neutral sphingomyelinase 2 inhibitor (PDDC) blocking maturation and full infectivity of HIV-1 particles	[38,39]
